# Population pharmacokinetics of piperacillin based on multicenter data to support model-informed precision dosing in critically ill women

**DOI:** 10.1128/aac.00986-26

**Published:** 2026-10-14

**Authors:** Sebastian Greppmair, Sina Pesthy, Michael Zoller, Christina Scharf, Alexander Brinkmann, Otto Frey, Johannes Zander, Nadine Liebchen, Stefan Hagel, Sebastian G. Wicha, Christoph Dorn, Sabrina Böing, Uwe Liebchen

**Affiliations:** 1 Department of Anaesthesiology, LMU University Hospital, LMU Munich 197746 , Munich, Germany; 2 Department of Anaesthesiology and Intensive Care Medicine, General Hospital of Heidenheim, Heidenheim, Germany; 3 Department of Pharmacy, General Hospital of Heidenheim, Heidenheim, Germany; 4 Laboratory Dr. Brunner, Laboratory Medical Care Center Konstanz GmbH, Constance, Germany; 5 Institute for Infectious Diseases and Infection Control, Jena University Hospital, Friedrich‐Schiller‐University Jena 9378, Jena, Germany; 6 Department of Clinical Pharmacy, Institute of Pharmacy, University of Hamburg 276709, Hamburg, Germany; 7 Department of Pharmaceutical and Medicinal Chemistry I, University of Regensburg 9147, Regensburg, Germany; 8 Doctoral Program Clinical Pharmacy, LMU University Hospital, LMU Munich 27192 , Munich, Germany; Providence Portland Medical Center, Portland, Oregon, USA

**Keywords:** sepsis, population pharmacokinetics, model-informed precision dosing, Bayesian forecasting, nonlinear mixed-effects modeling, critical care, sex-specific pharmacokinetics

## Abstract

Sex-specific differences in pharmacokinetics are increasingly recognized as clinically relevant, yet most population pharmacokinetic models for antibiotics have been developed in predominantly male populations with limited transferability to female patients. This study aimed to develop and externally evaluate a population pharmacokinetic model of piperacillin specifically for critically ill women to support model-informed precision dosing (MIPD). A total of 923 samples from 165 critically ill women (from four previously published studies) receiving piperacillin/tazobactam were analyzed. The data set was randomly split into a training set (80%) and an independent external evaluation set (20%). Non-linear mixed-effects modeling was employed based on the training data using NONMEM, and covariates were identified using stepwise covariate modeling with cross-validation. Prediction accuracy and bias were quantified using the median prediction error (MDPE) and the median absolute prediction error (MDAPE) in an MIPD scenario. Model predictions for future concentrations were generated both *a priori* and through Bayesian forecasting based on one prior sample. A two-compartment model provided the best fit for the data. Clearance was significantly affected by renal function estimated by the Cockcroft–Gault equation, along with procalcitonin and serum albumin. The external evaluation demonstrated strong predictive performance with very low systematic bias. In the MIPD scenario, *a priori* predictions exhibited good predictive performance (MDPE < |5.2|%, MDAPE < 33.4%), which substantially improved with Bayesian forecasting (MDPE < |3.2|%, MDAPE < 19.1%). A female-specific population pharmacokinetic model of piperacillin was developed and externally validated, demonstrating robust predictive performance and clear benefits for Bayesian forecasting.

## INTRODUCTION

Sex-related differences in pharmacokinetics (PK) are increasingly acknowledged as clinically relevant. Historically, pharmacological research has been predominantly conducted in male subjects, resulting in treatment guidelines that implicitly assume comparable PK across sexes (1). However, women differ from men in key physiological features affecting drug disposition, including lower body weight, higher body fat composition, and lower glomerular filtration rates (2). The incorporation of sex-based analyses in United States (U.S.) drug development was not mandatory until the Food and Drug Administration (FDA) implemented corresponding guidelines in 1998 (3). At equivalent dosing, women frequently exhibited higher plasma drug concentrations and prolonged elimination times, irrespective of body weight differences. Sex-related differences in PK strongly predicted adverse drug reactions (ADRs) in women, but not in men (4, 5). These findings indicate that clinically relevant sex differences in PK may be relevant yet remain underrepresented in current dosing strategies.

We recently evaluated model-informed precision dosing (MIPD) strategies for piperacillin (PIP), the most frequently used β-lactam antibiotic in critical care (6). MIPD integrates population PK models with individual patient characteristics and, when available, measured drug concentrations to guide individualized dosing decisions (7). In our multicenter analysis, we observed a systematic and clinically relevant prediction bias in female patients. Models with good overall performance demonstrated lower predictive accuracy in women compared with men, highlighting the need for sex-specific pharmacokinetic models for PIP model-based therapy. Notably, the majority of the investigated models were developed in predominantly male populations (6).

To date, only one population pharmacokinetic model of PIP has been developed specifically for critically ill women based on a monocentric cohort, including only 21 women (8). Therefore, we aim to develop and externally evaluate a PIP PK model optimized for MIPD based on a large, multicenter, cohort of critically ill women.

## MATERIALS AND METHODS

Reporting was guided by published guidelines for clinical pharmacokinetic studies (9).

### Data and patients

This population pharmacokinetic analysis was based on data derived from four previously published studies conducted in 11 German intensive care units. The original studies were previously published and collectively utilized in a model evaluation study (6, 10–13). All studies were approved by the respective institutional ethics committees and conducted in accordance with applicable ethical standards. For details, please refer to the individual studies.

For this analysis, only female patients without kidney replacement therapy were included. Clinical data from 165 critically ill women treated with piperacillin/tazobactam (PIP/TZP) were available, comprising 923 measured total PIP serum concentrations. Patients’ characteristics are provided in Table 1.

**TABLE 1:** Demographic and clinical characteristics of the study population stratified by study center

Characteristic	Overall *N* = 165^*a*^	Study center 1 (target study [13]) *N* = 55^*a*^	Study center 2 (Heidenheim [10]) *N* = 91^*a*^	Study center 3 (Regensburg [11]) *N* = 6^*a*^	Study center 4 (Munich [12]) *N* = 13^*a*^
Age [years]	71 (21–94)	68 (35–89)	73 (21–94)	60 (26–81)	70 (23–78)
Height [cm]	164 (135–178)	165 (153–178)	164 (135–175)	163 (158–170)	163 (150–176)
Weight [kg]	70 (43–193)	78 (43–193)	70 (44–170)	70 (55–100)	65 (50–100)
Ideal Body Weight [kg]	56 (30–69)	57 (46–69)	56 (30–66)	55 (51–61)	55 (4–67)
Creatinine clearance^*b*^ [mL/min]	51 (10–224)	53 (10–224)	50 (10–178)	45 (11–76)	63 (28–158)
Serum creatinine [mg/dL]	1.1 (0.3–4.4)	1.0 (0.3–4.4)	1.1 (0.4–3.8)	1.9 (1.1–4.0)	0.9 (0.6–1.8)
CRP [mg/dL]	13 (1–31)	13 (4–31)	NA^*c*^	10 (5–26)	16 (1–28)
Procalcitonin [ng/mL]	1.6 (0.1–247.3)	2.1 (0.1–247.3)	NA	0.3 (0.1–0.5)	NA
Serum albumin [g/dL]	2.5 (1.1–4.0)	2.6 (1.1–4.0)	NA	2.2 (1.8–2.5)	2.7 (2.2–3.4)
Sepsis	138 (84)	55 (100)	69 (76)	1 (17)	13 (100)
Total serum piperacillin concentration [mg/L]	71.4 (13.5–451.8)	78.8 (19.1–268.8)	66.5 (16.0–163.0)	78.2 (43.0–451.8)	98.0 (13.5–189.9)

^*a*^ Median (minimum–maximum); *n* (%).

^*b*^ According to the Cockcroft–Gault formula (14).

^*c*^ NA, not available.

PIP/TZP was administered in three studies intravenously with continuous infusion (CI), preceded by an optional loading dose at the physicians’ discretion, while one study employed intermittent infusion (II) over 30 min.

Blood samples for determination of total PIP plasma concentrations were collected at steady state. In the CI studies, samples were obtained at least once daily, while multiple samples at predefined time points during and after drug administration were collected in the II study. PIP concentrations were quantified using validated chromatographic or mass spectrometric methods. Information on concomitant medication was not systematically collected and, therefore, not included in the analysis.

### Training and evaluation data set

The full data set was randomly divided at the patient level into a training data set (80%) and an independent external evaluation data set (20%) without stratification by study center of infusion regimen.

Baseline demographic characteristics and relevant clinical covariates of the training and evaluation data set were comparable (see Tables S1 and S2).

### Pharmacokinetic modeling

Parametric population pharmacokinetic modeling was performed using the software NONMEM (version 7.5) based on the training data set following exploratory graphical analysis. One- and two-compartment models with linear elimination were evaluated. Stepwise covariate modeling (scm) with forward inclusion (*P* < 0.05) and backward elimination (*P* < 0.01) was applied to identify relevant covariates. At each step, covariate selection was guided by statistical significance, reduction in unexplained variability, precision of the estimated covariate effect, clinical relevance, and biological plausibility. After scm, cross-validated scm (xv-SCM, five groups, five splits) was performed to assess the robustness of covariate–parameter relationships. Further details regarding the modeling process are available in the Supplemental material 1.2. The NONMEM control stream of the final model is available in the Supplemental material 2.3.

### Model-informed precision dosing scenario

Both data sets (training and evaluation data set) were used to assess the predictive performance of the final population pharmacokinetic model for future measured concentrations. Predictive performance was assessed for the trough (II) or steady-state (CI) concentration of the second dosing occasion. Predictions were generated (i) based solely on patient characteristics (*a priori* prediction) and (ii) by incorporating the observed trough or steady state concentration from the first dosing occasion using Bayesian forecasting (Bayesian prediction).

All predictions were obtained without re-estimation of population parameters (NONMEM, MAXEVAL = 0) using MDV coding to control the inclusion of observations for Bayesian updating.

Prediction bias and accuracy were quantified using the median prediction error (MDPE) and the median absolute prediction error (MDAPE) expressed as percentages. MDPE < |20|% and MDAPE < 35% were defined as acceptable based on literature values (6, 15). Details can be found in the Supplemental material, equations (1-3) and (1.4).

## RESULTS

### Population pharmacokinetic model

Measured concentrations were adequately described by a two-compartment model with linear elimination. Compared with a one-compartment model, the two-compartment model substantially improved model fit (ΔOFV = −211; BIC 7094.2 vs. 7292.9) and provided a better graphical description of the data. The model included systemic clearance as a linear elimination term (CL), central and peripheral volumes of distribution (V₁ and V₂), as well as intercompartmental clearance (Q). Interindividual variability was supported for CL and V₁, but not retained for Q and V₂ due to poor identifiability and lack of meaningful improvement in model fit. Interoccasion variability was identified on CL. Residual unexplained variability was best described by a proportional error model.

Inclusion of allometric scaling for CL and V_1_ resulted in a significant improvement in model fit and parameter precision and was, therefore, retained in the final model. In contrast, implementation of allometric scaling for other parameters did not lead to further improvement and was not included in order to maintain model parsimony. Renal function quantified by creatinine clearance estimated using the Cockcroft–Gault equation was identified as a significant covariate on CL. During the respective covariate selection step, Cockcroft–Gault provided the largest reduction in OFV (ΔOFV = −214.21), while the best-performing CKD-EPI and Jelliffe models showed smaller OFV reductions of −212.0 and −199.9, respectively. In addition, procalcitonin and serum albumin concentrations were retained as clinically plausible and statistically significant covariates on CL. Inclusion of covariates resulted in a relevant reduction in unexplained interindividual variability (base model coefficient of variation [CV] [%] CL: 66.9; V_1_: 55.1, final model: CL 37.6, V_1_: 41.2) and an overall drop in OFV of 297. Xv-SCM confirmed all covariate relationships. The final equations for the PK parameters were:


$$\begin{array}{rl} & {CL}_{i}[L/h]=6.25\cdot {\frac{IBW}{56.45}}^{0.75}\cdot {\frac{GFRCG}{60.35}}^{0.64}\cdot {\frac{PCT}{2.05}}^{-0.18}\cdot {\frac{ALB}{2.5}}^{0.62}\cdot e^{ETA_{-}CLi}\\ & V_{1i}[L]=9.74\cdot {\frac{IBW}{56.45}}^{1}\cdot e^{ETA_{-}V1i}\\ & V_{2}[L]=9.63\\ & Q[L/h]=15.6 \end{array}$$

where CL represents clearance; V_1_ and V_2_ are the volume distributions of the central and peripheral compartments, respectively; Q is the intercompartmental clearance; IBW is ideal body weight; GFRCG is glomerular filtration rate estimated by the Cockcroft–Gault equation; PCT is procalcitonin; ALB is serum albumin; and ETA represents the individual (_*i*_) random effect for the respective parameter.

Parameter estimates were generally precise, indicating model robustness (Table 2). Shrinkage was low for CL (7%), indicating sufficient information in the data to support reliable estimation of individual clearance parameters. In contrast, higher shrinkage was observed for V1 (65%). This is likely attributable to the inclusion of CI data, where limited information on the distribution phase reduces the ability to inform volume-related parameters. Shrinkage associated with IOV (55%) was also relatively high. This can be explained by the (partly) sparse sampling per occasion, with often only a single observation available, limiting the information to estimate occasion-specific deviations.

**TABLE 2:** Estimated parameters of the population pharmacokinetic model of piperacillin in critically ill women (*n*_patients_ = 123)^*a*^

Parameter	Final model estimate (RSE, %)			Bootstrap median (95% CI)	
Fixed-effect parameters	Value			Value	
Clearance [L/h]	6.25	(3.6)		6.25	(5.84–6.73)
Central volume of distribution [L]	9.74	(17.8)		9.52	(7.61–12.47)
Peripheral volume of distribution [L]	9.63	(5.6)		9.81	(6.80–13.55)
Intercompartmental clearance [L/h]	15.6	(20.4)		15.4	(8.34–24.6)
COV_CL_CG	0.64	(6.5)		0.64	(0.54–0.72)
COV_CL_PCT	−0.18	(9.4)		−0.18	(-0.24 to −0.13)
COV_CL_ALB	0.62	(37.6)		0.63	(0.22–0.96)
Interindividual variability parameters (IIV)	SD		CV %	SD	
Central volume of distribution [L]	0.36	(7.2)	37.6	0.38	(0.08–0.56)
Clearance [L/h]	0.40	(32.9)	41.2	0.36	(0.30–0.42)
Interoccasion variability parameters (IOV)	SD			SD	
Clearance [L/h]	0.10	(18.9)	10.4	0.10	(0.05–0.13)
Residual variability parameters (RUV)	SD			SD	
Proportional	0.25	(1.7)		0.24	(0.20–0.29)

^*a*^ RSE, relative standard error; CI, confidence interval; SD, standard deviation; CV, coefficient of variation; COV_CL_CG, effect of estimated glomerular filtration rate according to the Cockroft–Gault formula on clearance; COV_CL_PCT, effect of procalcitonin concentration on clearance; COV_CL_ALB, effect of albumin concentration on clearance.

GOF plots for the training data set demonstrated strong concordance between observed concentrations and both population and individual predictions with no evidence of systematic bias (Fig. S1). Residual-based diagnostics confirmed the adequacy of the residual error model (Fig. S2). Prediction-corrected visual predictive checks and NPDEs showed that the observed concentrations were well captured by the simulated prediction intervals across the entire time range (Fig. S3 and S4).

### External model evaluation

External evaluation based on the independent evaluation data set confirmed the good predictive performance of the final population pharmacokinetic model. GOF and residual plots revealed close agreement between observed concentrations and model predictions, with no apparent bias across the concentration range (Fig. S5 and S6). Prediction-corrected visual predictive check and NPDE demonstrated good concordance between observed and simulated concentrations (Fig. S7 and S8).

### Model-informed precision dosing

Model predictions demonstrated low bias and high precision in both the training and external evaluation data sets. *A priori* predictions yielded an MDPE < |5.2|% and acceptable imprecision, with MDAPE values <33.4% across both data sets.

**Fig 1:**
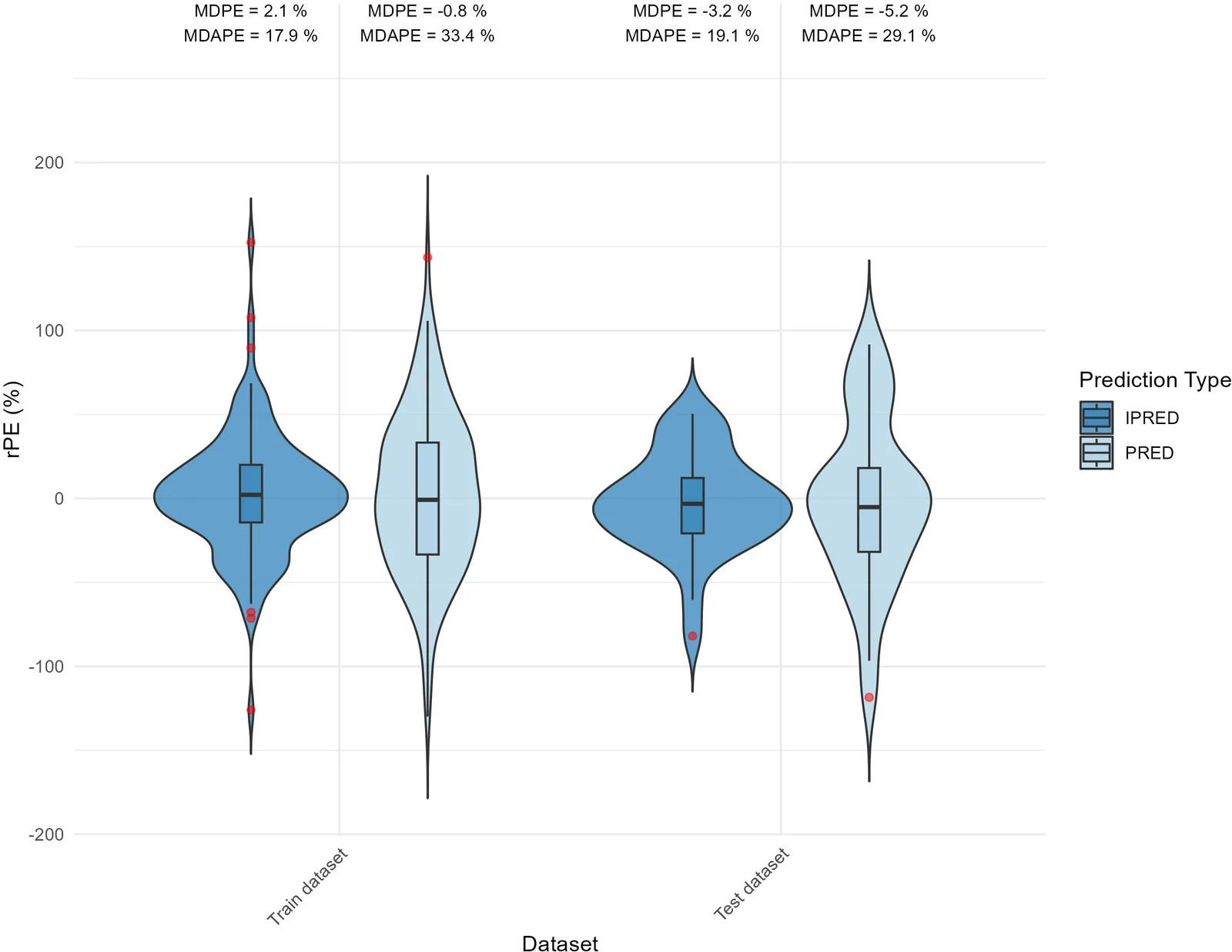
Distribution of relative prediction errors (PE) for population (PRED = *a priori*) and individual Bayesian predictions (IPRED = *a posteriori*) in the training and test data sets. Predictions are shown for the second observed piperacillin concentrations based on one integrated prior sample. Violin plots illustrate the full distribution of PE, with embedded boxplots indicating median and interquartile range. MDPE and MDAPE values are displayed above each panel as measures of bias and imprecision.

Bayesian forecasting, which incorporated the first observed concentration to predict the second concentration, substantially improved predictive performance. With Bayesian forecasting, MDPE values were <|3.2%|, while MDAPE values were <19.1%, indicating minimal systematic bias and imprecision within an acceptable range (Fig. 1).

## DISCUSSION

This is the first large, multicenter, externally evaluated population PK model of PIP specifically developed for critically ill women, directly addressing a documented source of systematic prediction bias in existing MIPD strategies (6).

In the past, numerous population pharmacokinetic models of PIP have been developed in critically ill patients. In 2023, our systematic literature review identified 24 parametric models (6). However, all of these models were based on mixed-sex populations of male and female patients, with a clear overrepresentation of male patients. In addition, while several additional PK models have been developed for specific subgroups, only one model by El-Haffaf et al. has been specifically developed for critically ill women (8). This study explored sex-specific covariate selection during population PK modeling of PIP and demonstrated that creatinine clearance and estimated glomerular filtration rate may differentially describe pharmacokinetic variability in male and female patients. Using a sex-stratified modeling approach in a relatively small cohort (21 female patients), the authors showed that dosing recommendations differed between sexes and that sex-specific covariate structures may improve predictive performance (8).

The present study substantially advances these findings by adopting an alternative modeling strategy. Instead of incorporating sex as a stratification factor within a mixed population, we developed a population pharmacokinetic model solely for critically ill women. Our approach and large data set enabled precise estimation of sex-specific structural pharmacokinetic parameters and allowed for robust external evaluation.

The majority of previously published PIP PK models relied on creatinine-based renal function estimates, most commonly Cockcroft–Gault (6). PIP is predominantly eliminated via the kidney, with approximately ~68–89% of the dose excreted unchanged through a combination of glomerular filtration and active tubular secretion (16). Active secretion is mediated by renal drug transporters, particularly OAT1 and OAT3, for which PIP acts as both a substrate and an inhibitor. Previous analyses suggest that OAT-mediated tubular secretion may contribute substantially to total clearance (in the range of ~50–74%), although exact quantitative contributions in humans remain uncertain (16, 17). In detail, OAT1 and OAT3 mediate active renal tubular secretion of PIP from blood into urine and, therefore, directly increase systemic drug clearance (18, 19). Sex-related differences in these transporters have been suggested mainly from preclinical data, but evidence in humans remains limited (20). Moreover, these OATs are strongly affected by systemic inflammation, with *in vivo* data indicating reductions in transporter activity of up to 40% under inflammatory conditions (21). In our model, the inclusion of procalcitonin as a covariate on clearance may, therefore, reflect inflammation-mediated suppression of tubular drug secretion. Low albumin concentrations likely reflect higher disease severity and capillary leakage rather than direct protein binding effects. Together, these covariates incorporate key aspects of sex-specific biology in critical illness into a PK model beyond simple adjustments for body size and renal function.

This analysis represents the largest population PK study of PIP conducted exclusively in women and one of the largest population PK analyses of PIP published to date. In contrast, most previously published PIP population PK models were generally developed in small, often monocentric cohorts with a median sample size typically around 20 patients (6, 22). The multicenter design and inclusion of both II and CI regimens increase the generalizability of the model across a wide range of ICU practice. The large data set enabled a robust methodological framework, including data set splitting with true external evaluation (132 patients for model development and 33 for external validation). In addition, cross-validated stepwise covariate modeling (xv-scm) was used to ensure stability of covariate relationships and minimize the risk of overfitting. The model was further evaluated in a MIPD framework using both *a priori* and Bayesian predictions, facilitating direct translation into clinical dosing workflows. Our model demonstrated superior predictive performance in the evaluation data set compared to all previously evaluated parametric models in a mixed population (6).

This analysis has the potential to substantially influence dosing strategies for PIP in clinical practice. MIPD offers substantial benefits, particularly for antibiotics with high PK variability (23, 24). PIP represents a paradigmatic example, as excessive exposure has been linked with nephrotoxicity and, when combined with vancomycin, an increased risk of acute kidney injury and potentially worse clinical outcomes (13, 18). Consequently, individualized dosing strategies are essential to ensure effective treatment of severe infections while minimizing avoidable toxicity. This study fills a gap in current clinical practice addressing approximately half of the patient population, for whom current dosing models systematically underperform. For clinical practice, our model offers a validated framework for more accurate model-informed precision dosing in women contributing to improved antibiotic stewardship by reducing unnecessary overexposure. Although external evaluation was performed within the same network of centers, independent validation in truly external cohorts is warranted prior to widespread implementation. Following such validation, integration into MIPD software facilitates routine clinical use.

More broadly, while differences in body composition are often viewed as the defining distinctions between men and women, our findings underscore the importance of sex-aware PK modeling as a prerequisite for equitable and safe precision dosing in critical care. Our findings may have implications for outcome disparities in critical care, even if our study was not designed to evaluate this aspect. Large observational studies have reported higher 30-day mortality in women with sepsis following ICU admission, with an absolute difference of approximately 3% (25, 26). These might be (partly) attributable to sex-indifferent dosing and thus might be overcome by sex-specific dosing.

Several limitations should be acknowledged. First, although the data set was multicenter, all participating ICUs were located in Germany, potentially limiting generalizability to other healthcare settings, even though no ethnic differences in PK are known for PIP (27). Second, patients receiving kidney replacement therapy or extracorporeal support were excluded, and, thus, the model should not be extrapolated to these populations. Third, only total PIP concentrations were analyzed, and variability in protein binding may influence the unbound drug exposure. Fourth, covariates were explored based on clinical relevance and data availability across the pooled multicenter data set. PCT and albumin were only available in a subset of studies (63 and 55% unavailability). These covariates were evaluated using the available data and retained in the model since they were physiologically plausible and significantly improved model fit and reduced unexplained variability. While median imputation enables model application when these covariates are unavailable, missing values may reduce the degree of individualization. Nevertheless, the model showed good overall predictive performance despite this limitation, including data from centers in which these covariates were unavailable.

### Conclusion

This study presents the first large, multicenter, externally validated population pharmacokinetic model of piperacillin specifically developed for critically ill women. Our model showed robust predictive performance with improved predictive performance using Bayesian forecasting. Our findings highlight the importance of sex-specific pharmacokinetic modeling as a prerequisite for accurate and equitable model-informed precision dosing in critical care.

## Data Availability

The data set is available upon request from the corresponding author.

## SUPPLEMENTAL MATERIAL

Supplemental material aac.00986-26-s0001.docx Tables S1 and S2; Figs. S1 to S8.

ASM does not own the copyrights to Supplemental Material that may be linked to, or accessed through, an article. The authors have granted ASM a non-exclusive, world-wide license to publish the Supplemental Material files. Please contact the corresponding author directly for reuse.
