# Supplementary material for: Population pharmacokinetics of piperacillin based on multicenter data to support model-informed precision dosing in critically ill women
Source: Antimicrob Agents Chemother. 2026 Oct 14;70(11):e00986-26. doi: 10.1128/aac.00986-26 (PMC13731236; doi:10.1128/aac.00986-26)
Supplement: Supplementary file 1 [file aac.00986-26-s0001.docx]

Supplementary materials

Category: Original Article

**Population pharmacokinetics of piperacillin based on multicenter data to support model-informed precision dosing in critically ill women**

Sebastian Greppmair^1^, Sina Pesthy^1^, Michael Zoller^1^, Christina Scharf^1^, Alexander Brinkmann^2^, Otto Frey^3^, Johannes Zander^4^, Nadine Liebchen^1^, Stefan Hagel^5^, Sebastian G. Wicha^6^, Christoph Dorn^7^, Sabrina Böing^1,8^, Uwe Liebchen^1,^*

^1^Department of Anaesthesiology, LMU University Hospital, LMU Munich, Germany

^2^ Department of Anaesthesiology and Intensive Care Medicine, General Hospital of Heidenheim, 89522 Heidenheim, Germany

^3^ Department of Pharmacy, General Hospital of Heidenheim, Heidenheim, Germany

^4^ Laboratory Dr. Brunner, Laboratory Medical Care Center Konstanz GmbH, Constance, Germany

^5^ Institute for Infectious Diseases and Infection Control, Jena University Hospital, Friedrich‐Schiller‐University Jena, Jena, Germany

^6^ Department of Clinical Pharmacy, Institute of Pharmacy, University of Hamburg, Germany

^7^ Department of Pharmaceutical and Medicinal Chemistry I, University of Regensburg, 93053 Regensburg, Germany.

^8^ Doctoral Program Clinical Pharmacy, LMU University Hospital, LMU Munich, Germany

*Corresponding author: Dr. med. Uwe Liebchen, Department of Anaesthesiology, LMU University Hospital, LMU Munich, Marchioninistrasse 15, 81377 Munich, Germany, Tel +49 89 4400 1684160, e‑mail: [uwe.liebchen@med.uni-muenchen.de](mailto:uwe.liebchen@med.uni-muenchen.de)

**Table of contents**

[1	Methods	3](#_Toc235523130)

[1.1	Training and evaluation datasets	3](#_Toc235523131)

[1.2	Details on pharmacokinetic modelling	4](#_Toc235523132)

[1.3	Software	5](#_Toc235523133)

[1.4	Calculation of prediction bias and imprecision	5](#_Toc235523134)

[2	Results	6](#_Toc235523135)

[2.1	Internal graphical model diagnostics	6](#_Toc235523136)

[2.2	External graphical model diagnostics	8](#_Toc235523137)

[2.3	Model code	13](#_Toc235523138)

[2.4	Stepwise covariate modelling	17](#_Toc235523139)

[2.4.1	Tested covariates	17](#_Toc235523140)

[2.4.2	SCM search	17](#_Toc235523141)

[2.5	References	18](#_Toc235523142)

# 1 Methods

## 1.1 Training and evaluation datasets

Table S1: Demographic and clinical characteristics of the training dataset stratified by study center; CRP, C-reactive protein.

| **Characteristic** | **Overall**, N = 132*^1^* | **Study center 1 (Jena**(1)**)**, N = 42*^1^* | **Study center 2 (Heidenheim**(2)**)**, N = 73*^1^* | **Study center 3 (Regensburg**(3)**)**, N = 5*^1^* | **Study center 4 (Munich**(4)**)**, N = 12*^1^* |
| --- | --- | --- | --- | --- | --- |
| **Age [years]** | 70 (21-94) | 69 (35-89) | 71 (21-94) | 62 (46-81) | 69 (23-78) |
| **Height [cm]** | 165 (135-176) | 165 (153-176) | 164 (135-175) | 160 (158-170) | 165 (150-176) |
| **Weight [kg]** | 73 (44-193) | 80 (47-193) | 70 (44-170) | 70 (55-100) | 64 (50-100) |
| **Ideal Body Weight [kg]** | 56 (30-67) | 57 (46-67) | 56 (30-66) | 52 (51-61) | 56 (43-67) |
| **Creatinine clearance [mL/min]** | 53 (10-224) | 48 (10-224) | 56 (13-178) | 27 (11-75) | 64 (28-158) |
| **Serum creatinine [mg/dL]** | 1.07 (0.29-4.41) | 1.05 (0.29-4.41) | 1.07 (0.35-3.77) | 2.29 (1.10-4.02) | 0.82 (0.60-1.70) |
| **CRP [mg/dL]** | 13 (1-31) | 17 (4-31) | NA | 12 (6-26) | 14 (1-28) |
| **Procalcitonin [ng/mL]** | 2.1 (0.1-84.0) | 2.8 (0.1-84.0) | NA | 0.3 (0.1-0.5) | NA |
| **Serum albumin [g/dL]** | 2.5 (1.2-4.0) | 2.6 (1.2-4.0) | NA | 2.1 (1.8-2.5) | 2.7 (2.2-3.4) |
| **Sepsis** | 109 (83) | 42 (100) | 54 (74) | 1 (20) | 12 (100) |
| **Total serum piperacillin concentration [mg/L]** | 71.2 (13.5-451.8) | 76.1 (19.1-268.8) | 66.5 (16.0-163.0) | 103.2 (43.0-451.8) | 92.1 (13.5-180.1) |
| *^1^*Median (Minimum-Maximum); n (%) | | | | | |

Table S2: Demographic and clinical characteristics of the evaluation dataset stratified by study center; CRP, C-reactive protein.

| **Characteristic** | **Overall**  N = 33*^1^* | **Study center 1 (Jena**(1)**)** N = 13*^1^* | **Study center 2 (Heidenheim**(2)**)**, N = 18*^1^* | **Study center 3 (Regensburg**(3)**)**, N = 1*^1^* | **Study center 4 (Munich**(4)**)**, N = 1*^1^* |
| --- | --- | --- | --- | --- | --- |
| **Age [years]** | 76 (26-91) | 63 (36-84) | 79 (48-91) | 26 (26-26) | 76 (76-76) |
| **Height [cm]** | 164 (150-178) | 165 (155-178) | 164 (150-172) | 170 (170-170) | 160 (160-160) |
| **Weight [kg]** | 70 (43-130) | 73 (43-130) | 70 (45-107) | 65 (65-65) | 70 (70-70) |
| **Ideal Body Weight [kg]** | 56 (43-69) | 57 (48-69) | 56 (43-63) | 61 (61-61) | 52 (52-52) |
| **Creatinine Clearance [mL/min]** | 50 (10-190) | 58 (24-190) | 34 (10-76) | 76 (76-76) | 29 (29-29) |
| **Serum creatinine [mg/dL]** | 1.2 (0.5-3.6) | 0.8 (0.5-3.1) | 1.5 (0.7-3.6) | 1.2 (1.2-1.2) | 1.8 (1.8-1.8) |
| **CRP [mg/dL]** | 10 (5-31) | 10 (6-31) | NA | 5 (5-5) | 23 (23-23) |
| **Procalcitonin [ng/mL]** | 0.4 (0.1-247.3) | 0.9 (0.1-247.3) | NA | 0.2 (0.2-0.2) | NA |
| **Serum albumin [g/dL]** | 2.4 (1.1-3.1) | 2.4 (1.1-3.1) | NA | 2.3 (2.3-2.3) | 2.7 (2.7-2.7) |
| **Total serum piperacillin concentration [mg/L]** | 74.5 (35.5-200.0) | 85.7 (42.7-200.0) | 65.3 (35.5-131.0) | 48.3 (48.3-48.3) | 189.9 (189.9-189.9) |
| *^1^*Median (Minimum-Maximum); n (%) | | | | | |

## 1.2 Details on pharmacokinetic modelling

Interindividual and interoccasion variability (occasion defined as treatment day) was explored assuming a log-normal distribution. Residual unexplained variability was assessed using additive, proportional, and combined error models. All models were fitted using the first-order conditional estimation method with interaction (FOCE+I).

Allometric scaling was tested, with clearance parameters scaled to ideal body weight, using a fixed exponent of 0.75 and volume parameters using an exponent of 1 (5).

Covariate selection was guided by exploratory data analysis and prior knowledge. Covariates were evaluated based on changes in the objective function value (OFV), physiological plausibility, and clinical relevance. Continuous covariates were linearly time-interpolated where appropriate, and relationships in the model were centered on the population median. Renal function was evaluated using different estimates of creatinine clearance and glomerular filtration rate, including the Cockcroft–Gault, CKD-EPI, and Jelliffe equations, which were compared as potential covariates on clearance (6–8). Categorical covariates were tested using additive and proportional shift models, while continuous covariates were evaluated using proportional, hockey-stick and power models. Stepwise covariate modeling (scm) with forward inclusion (p<0.05) and backward elimination (p<0.01) was applied to identify relevant covariates. At each step, covariate selection was guided by statistical significance, reduction in unexplained variability, precision of the estimated covariate effect, clinical relevance, and biological plausibility. After scm, cross-validated scm (xv-SCM, 5 groups, 5 splits) was performed to assess the robustness of covariate–parameter relationships. Missing covariate values were imputed to the median when necessary. Covariates with centre-specific availability were evaluated and retained only if their effects were robust and physiologically plausible.

Model adequacy was assessed using internal model diagnostic tools based on the model building dataset, including goodness-of-fit (GOF) plots comparing observed concentrations with population and individual predictions, as well as residual-based diagnostics. Predictive performance was further evaluated using prediction-corrected visual predictive checks (pcVPCs, n=500) and normalized prediction distribution errors (NPDEs) (9). Model robustness and parameter stability were examined using bootstrap (n=1000) .

External model performance was assessed based on the evaluation dataset using GOF plots, NPDEs and pcVPCs as recommended (10).

## 1.3 Software

Pharmacokinetic analyses were performed using NONMEM® (version 7.5) in conjunction with Pirana (version 25.7.1) and PsN (version 5.6.3) (11, 12). Data management, statistical analyses, and graphical representations were conducted using R (version 4.5.1) and RStudio® (version 2025.05.0).

## 1.4 Calculation of prediction bias and imprecision

Relative prediction errors (rPE), median prediction errors (MDPE) and median absolute prediction errors (MDAPE) were calculated according to the following formula:

${rPE}_{i,j}= \frac{C_{Pred,i,j}- C_{obs,i,j}}{(C_{Pred,i,j}+ C_{obs,i,j})/2}$	(Eq. 1)

$MDPE[\%]=median (\{{rPE}_{1,1}, \ldots,{rPE}_{i,j}\})*100$	(Eq. 2)

$MDAPE[\%]=median (\left\{ \left| {rPE}_{1,1} \right|, | \ldots\left| , \left| {rPE}_{i,j} \right| \right\} \right)*100$	(Eq. 3)

*i*: individual patient, *j*: measurement.

Relative prediction errors (rPE) were calculated using a symmetric formulation with the average of predicted and observed concentrations in the denominator. This approach was chosen to avoid implicitly treating either predicted or observed concentrations as a gold standard. In critically ill patients, both prediction and measurement are subject to relevant uncertainty, particularly due to imprecision in sampling times and analytical variability. By using the mean of predicted and observed values, the rPE yields identical absolute error magnitudes for over- and underprediction and remains invariant to proportional scaling of both values. This symmetric formulation has been used in previous pharmacometric studies and allows consistent interpretation of prediction errors independent of their direction (13–15).

# 2 Results

## 2.1 Internal graphical model diagnostics

Figure S1: Goodness-of-fit plots for the population pharmacokinetic model in the training dataset. Observed concentrations (DV) are shown versus population predictions (PRED) and individual predictions (IPRED) on linear (upper panels) and logarithmic scales (lower panels). The solid black line represents the line of identity, and the dashed red line indicates the linear regression fit. The grey shaded area represents the 95% confidence interval around the regression line.

Figure S2: Conditional weighted residuals versus population predictions for the final model in the training dataset. The red dashed line shows the LOESS trend and the grey line indicates zero.

Figure S3: Prediction-corrected visual predictive checks (pcVPCs) stratified by mode of administration. Left panel shows continuous infusion, and right panel shows intermittent infusion. Blue dots represent observed piperacillin concentrations, solid red lines indicate median observed concentrations, and dashed red lines denote the 2.5th and 97.5th percentiles of the observed data. Solid black lines represent the corresponding median model predictions, and dashed black lines represent the corresponding 2.5th and 97.5th percentiles of the simulated data. Shaded areas represent the 95% prediction intervals of the corresponding simulated percentiles.

Figure S4: Histogram of normalized prediction distribution errors (NPDE) for the training dataset, overlaid with the theoretical standard normal distribution (black) and the empirical density estimate (red).

## 2.2 External graphical model diagnostics

Figure S5: Goodness-of-fit plots for the population pharmacokinetic model in the evaluation dataset. Observed concentrations (DV) are shown versus population predictions (PRED) and individual predictions (IPRED) on linear (upper panels) and logarithmic scales (lower panels). The solid black line represents the line of identity, and the dashed red line indicates the linear regression fit. The grey shaded area represents the 95% confidence interval around the regression line.

Figure S6: Conditional weighted residuals versus population predictions for the final model in the training dataset. The red dashed line shows the LOESS trend and the grey line indicates zero.

Figure S7: Prediction-corrected visual predictive checks (pcVPCs) for continuous infusion (intermittent infusion with N=1 not shown). Blue dots represent observed piperacillin concentrations, solid red lines indicate median observed concentrations, and dashed red lines denote the 2.5th and 97.5th percentiles of the observed data. Solid black lines represent the corresponding median model predictions, and dashed black lines represent the corresponding 2.5th and 97.5th percentiles of the simulated data. Shaded areas represent the 95% prediction intervals of the corresponding simulated percentiles.

Figure S8: Histogram of normalized prediction distribution errors (NPDE) for the evaluation dataset, overlaid with the theoretical standard normal distribution (black) and the empirical density estimate (red).

## 2.3 Model code

;; Final population PK model of piperacillin in critically ill women

;; Two-compartment model with:

;; - IIV on CL and V1

;; - IOV on CL (occasion defined as day)

;; - Covariates on CL: GFRCG, PCT, ALBU

;; - Allometric scaling on CL and V1 using IBW

;; Residual error: proportional

;; -------------------------------------------------------------------

$PROBLEM Final covariate model: CL ~ IBW + GFRCG + PCT + ALBU with IOV

$INPUT ID TIME OCC EVID AMT DUR RATE CMT DV MDV SEX IBW GFRCG ALBU PCT TAD

$DATA	PIP_train.csv IGNORE=@

$SUBROUTINE ADVAN3 TRANS4

;=====================================================================

; PK MODEL

;=====================================================================

$PK

;--- Covariate effects on clearance -------------------------------

; Albumin effect

IF (ALBU.EQ.-99) THEN

CLALBU = 1

ELSE

CLALBU = (ALBU/2.5)**THETA(11)

ENDIF

; Procalcitonin effect

IF (PCT.EQ.-99) THEN

CLPCT = 1

ELSE

CLPCT = (PCT/2.05)**THETA(10)

ENDIF

; Renal function (Cockcroft–Gault)

CLGFRCG = (GFRCG/60.35)**THETA(9)

; Combined covariate effect on CL

CLCOV = CLGFRCG * CLPCT * CLALBU

;--- Allometric scaling --------------------------------------------

ALLOCL = (IBW/56.45)**THETA(8)

ALLOV = (IBW/56.45)**THETA(7)

;--- Interoccasion variability on CL -------------------------------

IF (OCC.EQ.1) IOVCL = ETA(4)

IF (OCC.EQ.2) IOVCL = ETA(5)

IF (OCC.EQ.3) IOVCL = ETA(6)

IF (OCC.EQ.4) IOVCL = ETA(7)

IF (OCC.EQ.5) IOVCL = ETA(8)

IF (OCC.EQ.6) IOVCL = ETA(9)

IF (OCC.EQ.7) IOVCL = ETA(10)

IF (OCC.EQ.8) IOVCL = ETA(11)

IF (OCC.EQ.9) IOVCL = ETA(12)

IF (OCC.EQ.10) IOVCL = ETA(13)

;--- Typical values ------------------------------------------------

TVCL = THETA(3) * ALLOCL * CLCOV

TVV1 = THETA(4) * ALLOV

TVV2 = THETA(5)

TVQ = THETA(6)

;--- Individual parameters ----------------------------------------

CL = TVCL * EXP(ETA(1) + IOVCL)

V1 = TVV1 * EXP(ETA(2))

V2 = TVV2 * EXP(ETA(3))

Q = TVQ

S1 = V1

S2 = V2

;=====================================================================

; ERROR MODEL

;=====================================================================

$ERROR

IPRED = F

DEL	= 0

IF (IPRED.EQ.0) DEL = 0.0001

; Combined error (effectively proportional)

W = SQRT((THETA(1)*IPRED)**2 + THETA(2)**2)

IRES = DV - IPRED

IWRES = IRES/(W+DEL)

Y	= IPRED + W*EPS(1)

;=====================================================================

; FIXED EFFECTS

;=====================================================================

$THETA

0.24633	; THETA(1): proportional error

0.0001 FIX	; THETA(2): additive error (fixed)

6.25386	; THETA(3): CL (L/h)

9.74448	; THETA(4): V1 (L)

9.62601	; THETA(5): V2 (L)

15.5533	; THETA(6): Q (L/h)

1 FIX	; THETA(7): allometry V

0.75 FIX	; THETA(8): allometry CL

0.637238; THETA(9): GFRCG effect

-0.178393 ; THETA(10): PCT effect

0.617027 ; THETA(11): ALBU effect

;=====================================================================

; RANDOM EFFECTS

;=====================================================================

$OMEGA

0.131646	; ETA(1): IIV on CL

0.156794	; ETA(2): IIV on V1

0 FIX	; ETA(3): no IIV on V2

; IOV on CL (one ETA per occasion)

$OMEGA BLOCK(1) 0.0107501

$OMEGA BLOCK(1) SAME

$OMEGA BLOCK(1) SAME

$OMEGA BLOCK(1) SAME

$OMEGA BLOCK(1) SAME

$OMEGA BLOCK(1) SAME

$OMEGA BLOCK(1) SAME

$OMEGA BLOCK(1) SAME

$OMEGA BLOCK(1) SAME

$OMEGA BLOCK(1) SAME

;=====================================================================

; RESIDUAL VARIABILITY

;=====================================================================

$SIGMA 1 FIX

;=====================================================================

; ESTIMATION

;=====================================================================

$ESTIMATION METHOD=1 INTER MAXEVAL=9999 NOABORT SIG=3 PRINT=1 POSTHOC

$COVARIANCE MATRIX=S PRINT=E

;=====================================================================

; OUTPUT TABLES

;=====================================================================

## 2.4 Stepwise covariate modelling

### 2.4.1 Tested covariates

| Covariate | Parameter |
| --- | --- |
| Creatinine clearance according to   - Cockcroft-Gault formula - Jelliffe formula - CKD-EPI formula | CL |
| Albumin | CL |
| APACHE II score | CL |
| SOFA score | CL |
| Bilirubin | CL |
| Cholinesterase | CL |
| Height | CL |
| Total body weight | CL |
| C-reactive protein | CL |
| Interleukin-6 | CL |
| Ideal body weight | CL |
| Body mass index | CL |
| Lean body mass | CL |
| Lactate | CL |
| Procalcitonin | CL |
| APACHE II | V1 |
| Height | V1 |
| Total body weight | V1 |
| Ideal Body weight | V1 |
| SOFA score | V1 |

### 2.4.2 SCM search

| Model | **2CMT + IIV&IOV CL + IIV V1** | **+ Allometric scaling (V1 + CL)** | **+COV_GFRCG_CL** | **+COV_PCT_CL** | **+COV_ALB_CL** |
| --- | --- | --- | --- | --- | --- |
| OFV (forward inclusion) | 6070.85 | 6062.60 (Δ-8.25) | 5848.39 (Δ-214.21) | 5784.52 (Δ-63.86) | **5773.85** (Δ-10.68) |
| Model | **Full forward** | **-COV_ALB_CL** | **-COV_PCT_CL** | **-COV_GFRCG_CL** |  |
| OFV (backward elimination) | **5773.85** | 5784.52 (Δ+10.67) | 5842.55 (Δ+68.70) | 5933.03 (Δ+159.18) |  |

## 2.5 References

1.	Hagel S, Bach F, Brenner T, Bracht H, Brinkmann A, Annecke T, Hohn A, Weigand M, Michels G, Kluge S, Nierhaus A, Jarczak D, König C, Weismann D, Frey O, Witzke D, Müller C, Bauer M, Kiehntopf M, Neugebauer S, Lehmann T, Roberts JA, Pletz MW, the TARGET Trial Investigators, Braune A, Schmidt K, Motsch J, Pinder N, Richter D, Schlattmann P, Ameln-Mayerhofer von A, Schappacher M, Fuchs T, Röhr A, Kurlbaum M, Schreiner O, Hüter L, Gründling M, Angermair S, Deja M, Bloos F, Fiedler S, Chkirni H. 2022. Effect of therapeutic drug monitoring-based dose optimization of piperacillin/tazobactam on sepsis-related organ dysfunction in patients with sepsis: a randomized controlled trial. Intensive Care Med 48:311–321.

2.	Richter DC, Frey O, Röhr A, Roberts JA, Köberer A, Fuchs T, Papadimas N, Heinzel-Gutenbrunner M, Brenner T, Lichtenstern C, Weigand MA, Brinkmann A. 2019. Therapeutic drug monitoring-guided continuous infusion of piperacillin/tazobactam significantly improves pharmacokinetic target attainment in critically ill patients: a retrospective analysis of four years of clinical experience. Infection 47:1001–1011.

3.	Schießer S, Hitzenbichler F, Kees MG, Kratzer A, Lubnow M, Salzberger B, Kees F, Dorn C. 2020. Measurement of Free Plasma Concentrations of Beta-Lactam Antibiotics: An Applicability Study in ICU Patients. Ther Drug Monit https://doi.org/10.1097/FTD.0000000000000827.

4.	Zander J, Döbbeler G, Nagel D, Maier B, Scharf C, Huseyn-Zada M, Jung J, Frey L, Vogeser M, Zoller M. 2016. Piperacillin concentration in relation to therapeutic range in critically ill patients – a prospective observational study. Critical Care 20:79.

5.	McCarron MM, Devine BJ. 1974. Clinical Pharmacy: Case Studies: Case Number 25 Gentamicin Therapy. Drug Intelligence & Clinical Pharmacy 8:650–655.

6.	Cockcroft DW, Gault MH. 1976. Prediction of creatinine clearance from serum creatinine. Nephron 16:31–41.

7.	Levey AS, Stevens LA, Schmid CH, Zhang YL, Castro AF, Feldman HI, Kusek JW, Eggers P, Van Lente F, Greene T, Coresh J, CKD-EPI (Chronic Kidney Disease Epidemiology Collaboration). 2009. A new equation to estimate glomerular filtration rate. Ann Intern Med 150:604–612.

8.	Jelliffe RW. 1973. Letter: Creatinine clearance: bedside estimate. Ann Intern Med 79:604–605.

9.	Bergstrand M, Hooker AC, Wallin JE, Karlsson MO. 2011. Prediction-corrected visual predictive checks for diagnosing nonlinear mixed-effects models. AAPS J 13:143–151.

10.	Cheng Y, Wang C-Y, Li Z-R, Pan Y, Liu M-B, Jiao Z. 2021. Can Population Pharmacokinetics of Antibiotics be Extrapolated? Implications of External Evaluations. Clin Pharmacokinet 60:53–68.

11.	Keizer RJ, Van Benten M, Beijnen JH, Schellens JHM, Huitema ADR. 2011. Piraña and PCluster: A modeling environment and cluster infrastructure for NONMEM. Computer Methods and Programs in Biomedicine 101:72–79.

12.	Lindbom L, Pihlgren P, Jonsson N. 2005. PsN-Toolkit—A collection of computer intensive statistical methods for non-linear mixed effect modeling using NONMEM. Computer Methods and Programs in Biomedicine 79:241–257.

13.	Broeker A, Nardecchia M, Klinker KP, Derendorf H, Day RO, Marriott DJ, Carland JE, Stocker SL, Wicha SG. 2019. Towards precision dosing of vancomycin: a systematic evaluation of pharmacometric models for Bayesian forecasting. Clinical Microbiology and Infection 25:1286.e1-1286.e7.

14.	Greppmair S, Brinkmann A, Roehr A, Frey O, Hagel S, Dorn C, Marsot A, El-Haffaf I, Zoller M, Saller T, Zander J, Schatz LM, Scharf C, Briegel J, Minichmayr IK, Wicha SG, Liebchen U. 2023. Towards model-informed precision dosing of piperacillin: multicenter systematic external evaluation of pharmacokinetic models in critically ill adults with a focus on Bayesian forecasting. Intensive Care Med https://doi.org/10.1007/s00134-023-07154-0.

15.	Schatz LM, Greppmair S, Kunzelmann AK, Starp J, Brinkmann A, Roehr A, Frey O, Hagel S, Dorn C, Zoller M, Scharf C, Wicha SG, Liebchen U. 2024. Predictive performance of multi-model approaches for model-informed precision dosing of piperacillin in critically ill patients. International Journal of Antimicrobial Agents 64:107305.
